# Linear chromosomes in bacteria: no straight edge advantage?

**DOI:** 10.1111/j.1462-2920.2007.01328.x

**Published:** 2007-05

**Authors:** Michael Y Galperin

**Affiliations:** National Center for Biotechnology Information, National Library of Medicine, National Institutes of Health Bethesda, MD 20894, USA

The beginning of 2007 brought us the complete genome of the yeast *Pichia stipitis,* five archaeal and more than 25 completely sequenced bacterial genomes (Table 1). In addition, there were two genomics-related papers that deserve a special discussion. One of them (Fuchs *et al*., 2007) described an unfinished genome of the ubiquitous marine phototrophic bacterium *Congregibacter litoralis*, while the other (Cui *et al*., 2007) examined the properties of the *Escherichia coli* K-12 strains with a linear chromosome.

**Table 1:** Recently completed microbial genomes (February–April 2007).

Species name	Taxonomy	GenBank accession	Genome size (bp)	Proteins (total)	Sequencing centre^a^	Reference
New organisms						
*Pichia stipitis*	*Eukaryota,* *Fungi*	CM000437, CP000496– CP000502	15 441 179(total)	5816	JGI	Jeffries *et al*. (2007)
*Pyrobaculum calidifontis*	*Crenarchaeota*	CP000561	2 009 313	2149	JGI	Unpublished
*Staphylothermus marinus*	*Crenarchaeota*	CP000575	1 570 485	1570	JGI	Unpublished
*Methanocorpusculum labreanum*	*Euryarchaeota*	CP000559	1 804 962	1739	JGI	Unpublished
*Methanoculleus marisnigri*	*Euryarchaeota*	CP000562	2 478 101	2489	JGI	Unpublished
*Mycobacterium* sp. JLS	*Actinobacteria*	CP000580	6 048 425	5739	JGI	Unpublished
*Saccharopolyspora erythraea*	*Actinobacteria*	AM420293	8 212 805	7197	University of Cambridge, UK	Oliynyk *et al*. (2007)
*Burkholderia vietnamiensis*	*β-Proteobacteria*	CP000614– CP000621	7 993 202 (total)	7617	JGI	Unpublished
*Herminiimonas arsenicoxydans*	*β-Proteobacteria*	CU207211	3 424 307	3325	Génoscope, Evry, France	Unpublished
*Acinetobacter baumannii*	*γ-Proteobacteria*	CP000521 CP000522 CP000523	3 976 747 13 408 11 302	3368	Yale University, New Haven, CT, USA	Smith *et al*. (2007)
*Actinobacillus pleuropneumoniae*	*γ-Proteobacteria*	CP000569	2 274 482	2012	NRC-Ottawa	Deslandes *et al*. (2007)
*Shewanella baltica*	*γ-Proteobacteria*	CP000563– CP000567	5 342 896 (total)	4489	JGI	Unpublished
*Shewanella loihica*	*γ-Proteobacteria*	CP000606	4 602 594	3859	JGI	Unpublished
*Clostridium difficile 630*	*Firmicutes*	AM180355 AM180356	4 290 252 7 881	3753	Sanger Institute	Sebaihia *et al*. (2006)
*Clostridium thermocellum*	*Firmicutes*	CP000568	3 843 301	3191	JGI	Unpublished
*Desulfotomaculum reducens*	*Firmicutes*	CP000612	3 608 104	3276	JGI	Unpublished
*Geobacillus thermodenitrificans*	*Firmicutes*	CP000557 CP000558	3 550 319 57 693	3392	Nankai University, Tianjin, China	Feng *et al*. (2007)
*Streptococcus sanguinis*	*Firmicutes*	CP000387	2 388 435	2270	VCU	Xu *et al*. (2007)
New strains						
*Methanococcus maripaludis* S5	*Euryarchaeota*	CP000609 CP000610	17 807 618 285	1822	JGI	Unpublished
*Prochlorococcus marinus* str. MIT 9301	*Cyanobacteria*	CP000576	1 641 879	1907	J. C. Venter Institute	Unpublished
*Corynebacterium glutamicum* R	*Actinobacteria*	AP009044 AP009045	3 314 179 49 120	3018	RITE	Yukawa *et al*. (2007)
*Rhodobacter sphaeroides* ATCC 17029	*α-Proteobacteria*	CP000577 CP000578 CP000579	3 147 721 1 219 053 122 606	4131	JGI	Unpublished
*Burkholderia mallei* NCTC 10229	*β-Proteobacteria*	CP000546 CP000545	3 458 208 2 284 095	5510	TIGR	Unpublished
*Burkholderia mallei* NCTC 10247	*β-Proteobacteria*	CP000548 CP000547	3 495 687 2 352 693	5852	TIGR	Unpublished
*Burkholderia pseudomallei* 668	*β-Proteobacteria*	CP000570 CP000571	3 912 947 3 127 456	7330	TIGR	Unpublished
*Burkholderia pseudomallei* 1106a	*β-Proteobacteria*	CP000572 CP000573	3 988 455 3 100 794	7183	TIGR	Unpublished
*Francisella tularensis* subsp. *tularensis*	*γ-Proteobacteria*	CP000608 CP000573	1 898 476	1634	JGI	Unpublished
WY96-3418 *Lactococcus lactis* subsp. *cremoris*MG1363	*Firmicutes*	AM406671	2 529 478	2434	Inst. Food Res., Norwich, UK	Wegmann *et al*. (2007)
*Streptococcus pyogenes* str. Manfredo	*Firmicutes*	AM295007	1 841 271	1746	Sanger Institute	Holden *et al*. (2007)

a Sequencing centre names are abbreviated as follows: JGI, US Department of Energy Joint Genome Institute, Walnut Creek, CA, USA; NRC-Ottawa, NRC Institute for Biological Sciences, Ottawa, Ontario, Canada; Sanger Institute, The Wellcome Trust Sanger Institute, Hinxton, Cambridge, UK; VCU, Virginia Commonwealth University, Richmond, VA, USA; RITE, Research Institute of Innovative Technology for the Earth, Soraku, Kyoto, Japan; TIGR, The Institute of Genomic Research, Rockville, Maryland, USA.

*Pichia stipitis*, the eighth yeast with a completely sequenced genome, has a remarkable capability to ferment xylose into ethanol under microaerophilic conditions, rapidly and with a high yield. This ability allows *P. stipitis* to degrade lignocellulose, a major side product of agricultural and timber industry, and makes it a very promising organism for production of biofuels. The genome sequence will provide new tools for metabolic engineering in *P. stipitis* and for engineering xylose metabolism in *Saccharomyces cerevisiae* (Jeffries *et al*., 2007).

Among the five newly sequenced archaeal genomes, the most interesting one is probably that of *Pyrobaculum calidifontis*, a facultatively aerobic, heterotrophic hyperthermophilic crenarchaeon originally isolated from a terrestrial hot spring in the Philippines. *Pyrobaculum calidifontis* grows optimally in aerobic conditions at 90–95°C and pH 7.0, using oxygen as terminal electron acceptor (Amo *et al*., 2002). It can also grow in anaerobic conditions, using nitrate (but not nitrite or sulfur-containing compounds such as elemental sulfur, thiosulfate, sulfate and sulfite) as terminal electron acceptor. Owing to the relative ease of handling, *P. calidifontis* could be a useful model organism for studying hyperthermophilic enzymes. In addition to *P. calidifontis* and two other *Pyrobaculum* species with completely sequenced genomes, *Pyrobaculum aerophilum* and *Pyrobaculum islandicum* (GenBank accession numbers AE009441 and CP000504 respectively), JGI scientists are currently sequencing the genome of *Pyrobaculum arsenaticum* and two closely related species, *Caldivirga maquilingensis* and *Thermoproteus neutrophilus*, see http://www.jgi.doe.gov/sequencing/why/CSP2006/hyperthermo.html.

Genomes of the four other archaea listed in Table 1, a heterotrophic sulfur-reducing hyperthermophilic crenarchaeon *Staphylothermus marinus*, two mesophilic members of the euryarchaeal order *Methanomicrobiales*, *Methanocorpusculum labreanum* and *Methanoculleus marisnigri*, and a new strain of *Methanococcus maripaludis,* have been sequenced primarily to widen the coverage of archaeal phylogenetic diversity for the purposes of comparative genomics.

In the previous column (Galperin, 2007), we discussed the diversity of mycobacteria, ranging from the well-known pathogenic species such as *Mycobacterium tuberculosis*, *Mycobacterium leprae* and *Mycobacterium bovis* to the soil isolates, capable of metabolizing various petroleum products. A detailed description of the genome of the BCG vaccine strain of *M. bovis*, mentioned in that column, has since been published (Brosch *et al*., 2007). It revealed a large number of gene duplications, gene losses and changes in gene expression levels compared with the parent wild-type strain of *M. bovis* (Brosch *et al*., 2007). Meanwhile, the list of soil mycobacteria with sequenced genomes has been further expanded by the addition of *Mycobacterium* sp. JLS, the third mycobacterial strain isolated from the creosote-polluted soil in Libby, Montana (Miller *et al*., 2004). Similarly to *Mycobacterium* sp. MCS and *Mycobacterium* sp. KMS whose genomes have been released earlier (GenBank accession numbers CP000384 and CP000518), this strain is capable of degrading polycyclic aromatic hydrocarbons.

There are two more actinobacterial genomes in the current list, both sequenced primarily because of their biotechnological potential. *Corynebacterium glutamicum* R is a strain of industrial interest that secretes glutamic acid and can be adapted for production of succinate and lactate (Inui *et al*., 2004). Although the genome itself is not much different from the previously sequenced genomes of other corynebacteria, its description (Yukawa *et al*., 2007) could be of general interest. First, it provides an excellent comparative analysis of all six corynebacterial genomes sequenced to date with particular emphasis on the distribution of sigma subunits of RNA polymerase, two-component signal transduction systems and the poorly characterized proteins. In addition, it offers a useful example of using comparative genomic data to improve our understanding of corynebacterial metabolism and the perspectives for metabolic engineering in these bacteria.

*Saccharopolyspora erythraea* (originally designated *Streptomyces erythraeus*) is a soil actinomycete that produces erythromycin A, a widely used macrolide antibiotic active against many Gram-positive bacteria. A detailed analysis of its genome (Oliynyk *et al*., 2007) revealed numerous genes for antibiotic resistance and for production of secondary metabolites, including siderophores, polyketides, terpenoids, carotenoids and non-ribosomally synthesized peptides. The genome sequence will now be used for the identification of the compounds produced by each separate polyketide synthase or non-ribosomal peptide synthetase and, it is to be hoped, for improvement of the existing industrial strains.

A remarkable property of the 8.2-Mb chromosome of *S. erythraea* was that, contrary to the expectations and the earlier data (Reeves *et al*., 1998), it proved to be circular. The chromosomes of its close relatives, *Streptomyces coelicolor* and *Streptomyces avermitilis*, are both linear, as is the chromosome of *Rhodococcus* sp. strain RHA1. These chromosomes are the largest in actinobacteria (8.7, 9.0 and 7.8 Mb respectively) and comprise some of longest DNA molecules in the prokaryotic world. In contrast, the shorter chromosomes of other actinobacteria are all circular. Hence, it was tempting to speculate that the linearity of streptomycetal chromosomes was somehow linked to and perhaps beneficial for the maintenance of these extremely long DNA molecules, even though it resulted in the instability of their termini (Chen *et al*., 2002). This idea has been contradicted by the discovery of equally long but circular chromosomes in the acidobacterium *Solibacter usitatus* (9.97 Mb), δ-proteobacterium *Myxococcus xanthus* (9.1 Mb), cyanobacterium *Trichodesmium erythraeum* (7.8 Mb), and, now, in the actinobacterium *S. erythraea.*

Linear chromosomes are also found in other bacterial lineages, including the pathogenic spirochaetes *Borrelia afzelii*, *Borrelia burgdorferi* and *Borrelia garinii* and the α-proteobacterium *Agrobacterium tumefaciens.* This makes the mechanisms of emergence and maintenance of linear replicons a very exciting area of research (Hinnebusch and Tilly, 1993; Volff and Altenbuchner, 2000; Bao and Cohen, 2003). An interesting insight into the properties of linear chromosomes came from the recent work of Japanese scientists, who deliberately linearized the chromosome of *E. coli* by using the telomeres and telomerase (TE1N) from the lambdoid phage N15 (Cui *et al*., 2007). Surprisingly, cells with linearized chromosomes were fully viable and did not differ from the wild type with respect to growth rates, cell and nucleoid morphologies or levels of gene expression of most genes. The genes encoding DNA gyrase (*gyrB*) and topoisomerase IV (*parC*, *parE*) were essential both for the wild type and for the strains with linearized chromosomes. In fact linear chromosome even offered certain advantages: *E. coli* strains with circular chromosomes whose chromosomal recombination was affected by a mutation in the XerCD recombinase or by a deletion of the *dif* site, exhibited much slower growth than the same mutants carrying linearized chromosomes (Cui *et al*., 2007). Given the recent suggestion that the *dif* site is the true termination site in *E. coli* genome (Hendrickson and Lawrence, 2007), it seems reasonable to conclude that a linear genome might have some advantages when it comes to the resolution of chromosome dimers. It remains to be seen whether this applies also to the long chromosomes of streptomycetes. In any case, analysis of linear chromosomes in bacteria, as well as of ring chromosomes in eukaryotes (Naito *et al*., 1998) remains a very intriguing topic.

Returning to the recently completed genomic sequences, *Herminiimonas arsenicoxydans* (also referred to as strain ULPAs1 or *Caenibacter arsenoxydans*) is a recently characterized β-proteobacterium that belongs to the family *Oxalobacteraceae* in the order *Burkholderiales* (Muller *et al*., 2006). It has been isolated at an industrial wastewater treatment plant from active sludge heavily contaminated with arsenic. This strain tolerates up to 5 mM arsenite [As(III)], oxidizing it to arsenate [As(V)] by means of a periplasmic arsenate reductase. It was also resistant to such heavy metals as Se(IV), Mn(II), Cr(III), Cd(II), Sb(III) and Ni(II) (Muller *et al*., 2003). Even before the completion of the genome, the response of *H. arsenicoxydans* to arsenic stress was analysed by proteomic approaches (Carapito *et al*., 2006).

Among the recently sequenced γ-proteobacterial genomes, *Acinetobacter baumannii* stands out because of its rapid evolution from a relatively benign environmental microorganism into a dangerous pathogen. This organism is a member of the family *Moraxellaceae* in the order *Pseudomonadaceae*, which is commonly found in soil and water samples and sometimes colonizes human skin and respiratory tract. However, in the course of the past three decades, *A. baumannii* gradually gained resistance to most commonly used antibiotics (Fournier *et al*., 2006) and became a widespread source of hospital-acquired infections, such as pneumonia, meningitis, bacteraemia and septicaemia. These infections result in high mortality, estimated to reach 30% in patients with heavy burns and even higher in patients with respiratory infections or septicaemia (Smith *et al*., 2007; Trottier *et al*., 2007). The sequenced strain *A. baumannii* ATCC 17978 was isolated from a patient with meningitis. A comparison of its genome to the previously sequenced genome of *Acinetobacter baylyi* strain ADP1 (Barbe *et al*., 2004) revealed the absence of certain catabolic genes and the presence of a large number of genes potentially involved in drug resistance or otherwise related to virulence. Many of the latter genes were clustered in 28 genomic islands that were likely acquired through a relatively recent lateral gene transfer (Smith *et al*., 2007). It is worth noting that *A. baumannii* genome was sequenced using a novel sequencing strategy, referred to as high-density pyrosequencing. This approach relies on clonal amplification of genomic DNA fragments, followed by parallel sequencing of numerous short (∼100 bp) DNA sequences, which makes their subsequent assembly quite a challenge. For this reason, pyrosequencing has been previously used for resequencing but not for *de novo* genome sequencing. These limitations were overcome by a combination of paired-end sequencing and polymerase chain reaction from the ends of contigs. The authors conclude that high-density pyrosequencing is a rapid method of sequencing that avoids the labour and potential bias of cloning steps. Future will show whether this method will prove cost-efficient for *de novo* genome sequencing.

The *Shewanella* genome sequencing project released complete genomes of two more strains. *Shewanella baltica*, isolated, as the name implies, from the Baltic Sea, is unusual in its ability to grow at 4°C, but not at 37°C. *Shewanella loihica* strain PV-4 was isolated from an iron-rich microbial mat next to a hydrothermal vent on the Loihi Seamount, Hawaii, at the depth of 1325 m. This strain is psychrotolerant with the optimal growth temperature of 18°C, but capable of surviving at temperatures from 0 to 45°C.

Although not assembled into a single contig (and hence not listed in Table 1), the genome of the marine γ-proteobacterium *Congregibacter litoralis* (Fuchs *et al*., 2007) is an important step towards understanding the bacterial diversity in the open sea. *Congregibacter litoralis* (formerly known as strain KT71) is a representative of the abundant γ-proteobacterial clade NOR5/OM60, originally isolated from the surface water in the North Sea (Eilers *et al*., 2001) and later found in marine bacterioplankton around the world. This organism carries a complete set of genes for the photosynthetic reaction centre, as well as genes coding for the biosynthesis of bacteriochlorophyll a and carotenoids. Accordingly, *C. litoralis* is capable of aerobic anoxygenic photosynthesis. However, it cannot grow photoautotrophically because it is incapable of autotrophic CO_2_ fixation and needs organic substrates such as carboxylic acids, oligopeptides, or fatty acids (Fuchs *et al*., 2007). It also requires oxygen and grows best in microaerophilic conditions. The genome of *C. litoralis* provides a window into the physiology of aerobic anoxygenic phototrophs, which during summer months may comprise up to 10% of the total marine bacterioplankton (Beja *et al*., 2002). This genome, deposited in the GenBank whole-genome division with accession no. AAOA00000000, currently consists of 42 contigs with a total of 4 325 534 bp, encoding 3950 proteins. The genome description itself (Fuchs *et al*., 2007) should become freely available in PNAS online in late August. Meanwhile, a somewhat lighthearted summary, comparing mixed energy requirements of *C. litoralis* with that of a hybrid car engine, is available on the MPI-Bremen web site, http://www.mpi-bremen.de/en/Marine_Bacteria_with_a_Hybrid_Engine.html.

The genome of *Clostridium difficile* strain 630, a major nosocomial pathogen that causes a variety of life-threatening gastrointestinal diseases (Cloud and Kelly, 2007), was sequenced at the Sanger Institute about a year ago (Sebaihia *et al*., 2006) but inadvertently missed in this column. The genome entry has been recently updated and is now listed in Table 1, just to ensure that it does not go unnoticed any longer.

Three other members of *Firmicutes* with the recently sequenced genomes are all environmental strains of biotechnological interest. *Clostridium thermocellum* is a moderately thermophilic anaerobe that secretes various cellulases and is capable of digesting extracellular cellulose. Its ability of converting cellulose-containing substrates directly into ethanol makes it an attractive candidate for production of biofuel from sawdust (Demain *et al*., 2005). While clostridial cellulases and the organization of the cellulosome have been studied for many years (Gilbert, 2007), the genome sequence will open new perspectives for metabolic engineering in this organism. *Desulfotomaculum reducens*, also a member of *Clostridiales*, is a mesophilic anaerobe, isolated from a heavy metal-contaminated sediment and capable of reduce uranium and chromium. It could be used for bioremediation of heavy metals.

*Geobacillus thermodenitrificans* strain NG80-2, isolated from a deep-subsurface oil reservoir in Dagang oilfield, Northern China, is moderately thermophilic (optimum 65°C) facultatively aerobic soil bacterium that can use long-chain (C_15_–C_36_) alkanes as a sole carbon source (Wang *et al*., 2006). The genome sequence revealed numerous adaptations to metabolism of various petroleum products, including a flavin-dependent monooxygenase that is capable of oxidizing long-chain alkanes to corresponding primary alcohols (Feng *et al*., 2007). *Geobacillus thermodenitrificans* encodes nitrous oxide reductase, which allows it to effectively use nitrate as terminal electron acceptor, reducing it to molecular nitrogen. The authors suggest that *G. thermodenitrificans* strain NG80-2 could be a good candidate for treatment of environmental oil pollutions and oily waste.

The list of recently sequenced microbial genomes also includes five genomes of *Burkholderia* spp., one from *Burkholderia vietnamiensis* and two each from *Burkholderia mallei and Burkholderia pseudomallei*; a genome of a new strain of *Prochlorococcus* sp., isolated from the Sargasso Sea at the depth of 90 m; genome of the swine pathogen *Actinobacillus pleuropneumoniae*; and the genomes of pathogenic strains of *Francisella tularensis*, *Streptococcus sanguinis* and *Streptococcus pyogenes* (Table 1).

One final note: while the description of the BCG genome (Brosch *et al*., 2007), mentioned above, was published shortly after its deposition in GenBank, many other bacterial genomes, particularly those sequenced at the JGI, are submitted to the public databases (GenBank/EMBL/DDBJ) immediately after completion and released to the public long before publication of the manuscripts describing these genomes. Since this column tracks the release of genomic sequences, rather than publication of the corresponding manuscripts, many sequenced genomes get listed as ‘unpublished’ (see Table 1). However, several months – or even years – later many genome descriptions do appear in press. For example, just in the past couple of months there appeared belated papers describing the complete genomes of avian pathogenic *E. coli* strain O1:K1:H7 (Johnson *et al*., 2007), *Bacillus thuringiensis* Al Hakam (Challacombe *et al*., 2007), *Mycobacterium ulcerans* (Stinear *et al*., 2007) and *Thermobifida fusca* (Lykidis *et al*., 2007). While some of these references have been included into the corresponding GenBank entries, others were not. Therefore, listing of a genome in Table 1 as ‘unpublished’ and absence of a linked reference in the GenBank file should both be taken with a grain of salt: there is always a chance that the genome description has been published.
